# Plant Oil-Based Acrylic Latexes towards Multisubstrate Bonding Adhesives Applications

**DOI:** 10.3390/molecules27165170

**Published:** 2022-08-13

**Authors:** Vasylyna Kirianchuk, Bohdan Domnich, Zoriana Demchuk, Iryna Bon, Svitlana Trotsenko, Oleh Shevchuk, Ghasideh Pourhashem, Andriy Voronov

**Affiliations:** 1 Department of Organic Chemistry, Institute of Chemistry and Chemical Technologies, Lviv Polytechnic National University, 79013 Lviv, Ukraine; 2 Department of Coatings and Polymeric Materials, North Dakota State University, Fargo, ND 58102, USA; 3 Oak Ridge National Laboratory, Chemical Sciences Division, Oak Ridge, TN 37830, USA

**Keywords:** plant oils, plant oil-based acrylic monomers, miniemulsion polymerization, biobased latexes, waterborne contact adhesive

## Abstract

To investigate the utility of acrylic monomers from various plant oils in adhesives manufacturing, 25–45 wt. % of high oleic soybean oil-based monomer (HOSBM) was copolymerized in a miniemulsion with commercially applied butyl acrylate (BA), methyl methacrylate (MMA), or styrene (St). The compositions of the resulting ternary latex copolymers were varied in terms of both “soft” (HOSBM, BA) and “rigid” (MMA or St) macromolecular fragments, while total monomer conversion and molecular weight of copolymers were determined after synthesis. For most latexes, results indicated the presence of lower and higher molecular weight fractions, which is beneficial for the material adhesive performance. To correlate surface properties and adhesive performance of HOSBM-based copolymer latexes, contact angle hysteresis (using water as a contact liquid) for each latex-substrate pair was first determined. The data showed that plant oil-based latexes exhibit a clear ability to spread and adhere once applied on the surface of materials differing by polarities, such as semicrystalline polyethylene terephthalate (PET), polypropylene (PP), bleached paperboard (uncoated), and tops coated with a clay mineral paperboard. The effectiveness of plant oil-based ternary latexes as adhesives was demonstrated on PET to PP and coated to uncoated paperboard substrates. As a result, the latexes with high biobased content developed in this study provide promising adhesive performance, causing substrate failure instead of cohesive/adhesive break in many experiments.

## 1. Introduction

It has become evident that the synthesis of sustainable polymers (based on natural resources) has developed into a booming research area that targets the replacing of petroleum-based counterparts in manufacturing with a broad range of polymeric materials. Renewable raw materials, such as cellulose, lignin, vegetable oils, starches, mono- and di-saccharides have attracted growing attention from both industrial and academic researchers in biobased polymeric material design [1,2]. Among others, plant/vegetable oils have become a prospective natural feedstock for synthesizing various biobased polymers and polymeric materials [3]. Chemically, they are mixtures of triglycerides, glycerol esters and various fatty acids. Depending on the oil composition, chain length, unsaturation degree, and types of fatty acids, moieties differ, essentially thus determining the physico-chemical properties of plant oils, as well as the synthesis possibilities and the prospects of particular oils. In fact, their chemical diversity is exciting, as well as offering various synthetic opportunities. Most chemical reactions of plant oil triglycerides proceed by reactions of the ester group, while some other synthetic pathways include reactions of allyl fragments [4]. When various other synthetic methods were applied, oxy-polymerized oils, polyesters [5], polyurethanes [6], polyamides, acrylates, and epoxy resins based on plant oil triglycerides [7] were successfully synthesized.

In the field of adhesives, polymeric materials from biobased renewable resources are increasingly being considered [8,9]. While using biopolymers, such as natural rubber, proteins, and polysaccharides (cellulose and starch, in particular) has been historically introduced, research interests in synthesizing polymeric adhesives, based on new renewable monomers, from various natural resources have recently grown, due to expansion in the sustainable products market. However, popular renewable feedstocks, like proteins, tend to increase the hydrophilicity of adhesives, due to the many polar groups in their structure, which creates a barrier to their incorporation in adhesive formulations. The use of plant-based polymeric materials can bring advantages to adhesives that were not previously possible [10]. Incorporated in adhesives formulation, structural elements from plant oils can increase adhesive hydrophobicity and, thus, water resistance [11,12,13]. Aside from hydrophobicity, polymer fragments derived from vegetable/plant oils can bring other advantages, such as plasticizing effects, compatibility between reagents, cross-linking sites, [14,15], etc. While the market for biobased adhesives is still limited, their advantages can be leveraged in different areas [16,17]. Moreover, using plant oil-based adhesives can improve the curing process, and strengthen adhesive bonds, while improving material sustainability by simplification of the recycling process, due to their inherent biodegradability, which saves efforts and costs, in terms of health and safety regulations.

Sustainability, however, is often not the only sufficient decision-making factor for commercializing biobased products. Modern chemical technologies should also enhance the performance compared to petroleum-based materials, especially if additional costs are required for new material implementation.

Synthesized in our group, plant oil-based monomers (POBMs) can be applied directly in the production of biobased polymeric materials that utilize acrylic monomers in free radical polymerization mechanisms, including emulsion/miniemulsion processes to yield latex polymers [18,19]. POBMs undergo free radical polymerization and, at the same time, retain reactive sites for post-polymerization cross-linking to generate materials with advanced properties and long-term performance. Depending on the applied oil(s), the chemical composition properties of POBM-based polymers can be tuned, based on the unsaturation amount of the fatty acid side chains [20,21,22]. The high functionality of POBM molecules facilitates control of the resulting polymer molecular weight and may enhance adhesion to a variety of substrates. Feasibility of synthesizing up to 70 wt. % plant oil-based cross-linkable latex binary copolymers has been demonstrated by our groups [20,21]. Such cross-linking may improve the mechanical properties and bonding strength of the resulting adhesives.

It is important to note that, despite the fact that plant oils are considered as a promising sustainable feedstock in biobased adhesive manufacturing, direct comparison of such adhesives with materials based on POBMs is not possible without an additional feasibility study which we report on in this publication.

This study focuses on synthesizing and characterizing novel plant oil-based ternary latex copolymers and evaluating their feasibility as adhesives on various substrates. For this purpose, a range of copolymers with high POBM content (25–45 wt. %) were synthesized using miniemulsion polymerization. To investigate the potential of POBMs as petroleum-based counterparts’ replacement in adhesives manufacturing, a high oleic soybean oil-based monomer (HOSBM) was chosen for copolymerization with the following substances, common in commercial latex adhesives: butyl acrylate (BA), methyl methacrylate (MMA), and styrene (St). We varied copolymer composition [including plant oil-based content and ratio between “soft” (POBMs, BA) and “rigid” (MMA or St) macromolecular fragments, based on merit] of the latex copolymers, while total monomer conversion, and particle size, as well as thermal characteristics of latexes, were considered as synthetic criteria and monitored after completion of polymerization.

Furthermore, the adhesive properties of the synthesized ternary latexes were evaluated using various substrate materials. As a result, newly developed POBM-based polymeric materials provide equivalent and, in some cases, better adhesive performance, causing substrate failure in many experiments. The utility of POBM-based ternary latexes as adhesives was clearly demonstrated on all substrates.

## 2. Results and Discussion

### 2.1. Synthesis and Characterization of Plant Oil-Based Terpolymers

For the synthesis of ternary latex copolymers, the concentration of plant oil-based fragments of HOSBM in monomer feed was varied (up to 45 wt. %) in copolymerization with MMA/St, while the content of the other (soft) counterpart, n-butyl acrylate, was held at 10 wt. %.

Miniemulsion copolymerization was completed within 10 h, during which essentially all monomers were polymerized (total monomer conversion of 90–95%) to yield thermal stable latexes at 30 wt. % solid content (Table 1).

The polymer composition is a significant parameter determining the properties of the terpolymer and, ultimately, its practical applications. The Alfrey–Goldfinger equations were used to estimate the compositions of the biobased terpolymers [23,24]. The resulting copolymers’ compositions were determined by ^1^H NMR spectroscopy, which is routinely used for the characterization of multicomponent polymers. The technique works best when the individual monomers exhibit well-defined signals unique to the specific monomer so that the accuracy of the signal integration is reliable. However, some uncertainty may arise when signals for individual monomers overlap. According to data in the literature for individual monomers ^1^H NMR spectra integrals, the molar fraction of BA/MMA/St, can be evaluated from ^1^H NMR-based integrated areas for the signals designated on the spectrum for -O-**CH_2_**- of BA (at 4.1–3.8 ppm, 2H), for -O-**CH_3_** of MMA (at 3.75–3.3 ppm, 3H), and for “aromatic hydrogens“ of St (spectral area for 6.2–7.5 ppm, 5H) [25].

**Figure 1:**
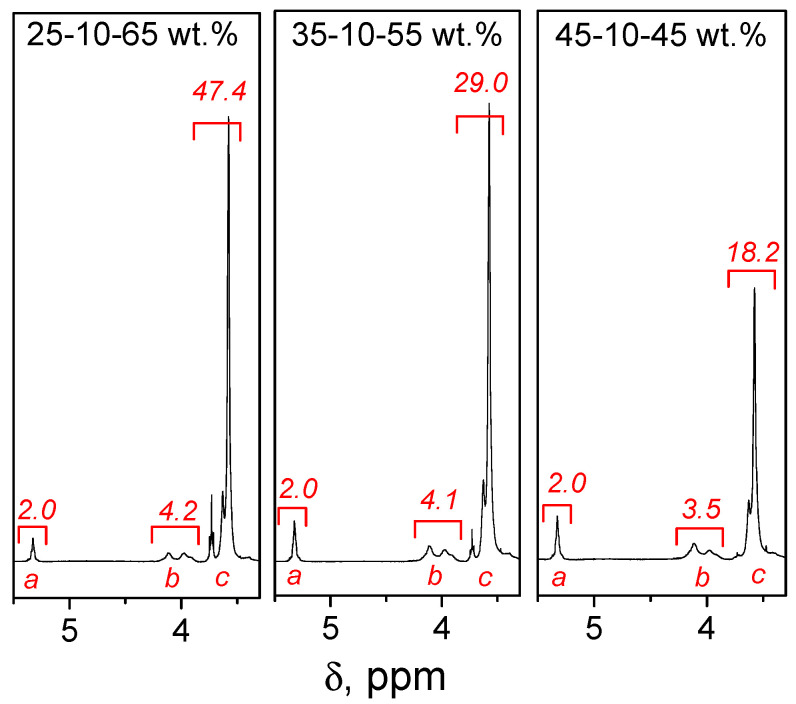
^1^H NMR spectra of terpolymers HOSBM-BA-MMA.

The ^1^H NMR spectrum of purified terpolymer HOSBM-BA-MMA (Figure 1) indicates the incorporation of all three monomer fragments through the appearance of characteristic signals for the protons of -**CH**=**CH**- (HOSBM), -O-**CH_3_** (MMA), and -O-**CH_2_**- (BA) groups at 5.37 (peak *a*), 4.15 (peak *b*), and 3.7 ppm (peak *c*), respectively. However, calculations of the composition for HOSBM-BA-MMA terpolymer are complicated, since the -O-**CH_2_**- signals of BA at 4.15 ppm overlap with HOSBM signals -O-**CH_2_**-CH_2_- at 4.2 ppm, as well as the -O-**CH_3_** signals of MMA at 3.75 ppm overlap with HOSBM signals -CH_2_-**CH_2_**-NH- at 3.6 ppm (Figure S1). To overcome this challenge, the ^1^H NMR spectrum of HOSBM homopolymer was used for calculations.

The resulting molar composition of terpolymers, based on integrals of characteristic peaks (*I_a_, I_b_, I_c_*) (Figure 1), was calculated using Equation (1). The amount of BA/MMA was calculated by subtracting the integral value of the signals at HOSBM homopolymer spectrum at ~4.15 and 3.7 ppm (2.2 and 2.3, respectively) from the determined value. The ^1^H NMR spectrum for HOSBM homopolymer is presented in the supporting information (Figure S2). The conversion calculations from mole fraction to weight fractions have been performed for all compositions presented in the Table 1. Terpolymers HOSBM-BA-MMA were obtained with high conversion (>90%), and their compositions based on ^1^H NMR measurements were very similar to the theoretical one (Table 1).

(1) $$\left[HOSBM\right]:\left[BA\right]:\left[MMA\right]=\frac{I_{a}/2}{I_{t}}:\frac{I_{b}/2}{I_{t}}:\frac{I_{c}/3}{I_{t}}$$

(2) $$I_{t}=\frac{I_{a}}{2}+\frac{I_{b}}{2}+\frac{I_{c}}{3}$$

**Figure 2:**
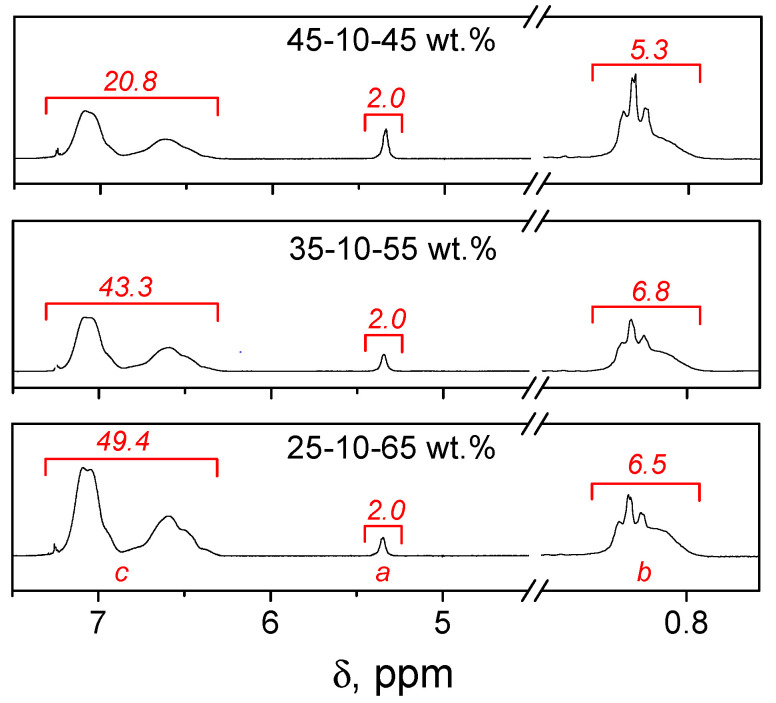
^1^H NMR spectra of terpolymers HOSBM-BA-St.

The ^1^H NMR spectrum of purified terpolymer HOSBM-BA-St (Figure 2) indicated the incorporation of St units through the appearance of characteristic signals for the aromatic hydrogens at 6.2–7.5 ppm (peak *c*). The characteristic signals for the protons of -**CH**=**CH**- group (HOSBM) appeared at 5.37 ppm (peak *a*). Since the -CH_2_-**CH_3_** signals of BA at 0.96 ppm (peak *b*) overlapped with HOSBM signals -CH_2_-**CH_3_** at 0.9 ppm the ^1^H NMR spectrum of the HOSBM homopolymer was used to calculate the composition for HOSBM-BA-St terpolymers. The molar composition of terpolymers was determined via ^1^H NMR spectra based on integrals of these characteristic peaks (*I_a_, I_b_, I_c_*) using Equation (3). The weight fractions were calculated from the mole fraction and presented in Table 1. The amount of BA was calculated by subtracting the integral value of the signals at HOSBM homopolymer spectrum at ~0.9 (3.2) from the determined value.

(3) $$\left[HOSBM\right]:\left[BA\right]:\left[St\right]=\frac{I_{a}/2}{I_{t}}:\frac{I_{b}/3}{I_{t}}:\frac{I_{c}/5}{I_{t}}$$

(4) $$I_{t}=\frac{I_{a}}{2}+\frac{I_{b}}{3}+\frac{I_{c}}{5}$$

Overall, obtained data on biobased content in the resulting latexes confirmed that the vast majority of plant oil-based ingredients were incorporated into the copolymer macromolecules during miniemulsion polymerization, and the composition of copolymers determined using NMR data coincided well with the composition calculated by the Alfrey–Goldfinger equations.

**Figure 3:**
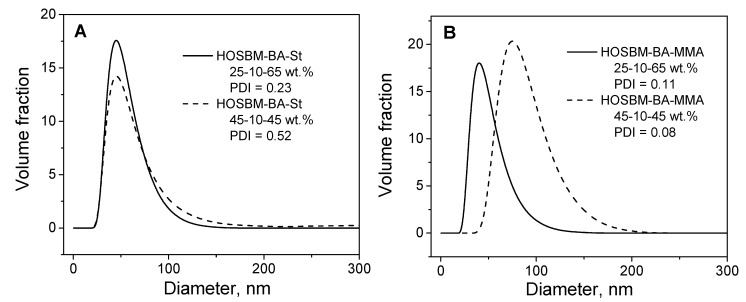
Latex particle size distribution of the biobased terpolymers HOSBM-BA-St (**A**) and HOSBM-BA-MMA (**B**).

The mean particle size distribution of the latex polymer particles was determined using dynamic light scattering. Figure 3 shows that the volume-average particle size for all terpolymer latexes ranged between 43 and 82 nm, while particle size distribution increased with increasing HOSBM content in monomer feed (PDI = 0.08–0.52).

Polymer molecular weight and dispersity are important parameters impacting adhesive performance. It is not uncommon that these parameters may have a complex influence on different properties. For example, good shear adhesion requires high molecular weight polymers with high entanglement molecular weight. The segment molecular weight between the crosslink points can be a factor if the formation of the crosslinked adhesive network is induced. On the other hand, to ensure good tackiness, macromolecules with lower molecular weight (and Tg) are required. Thus, polymers with bimodal (lower and higher fractions simultaneously) molecular weight distribution may facilitate enhanced values of both shear adhesion and tackiness of the adhesive [26].

**Figure 4:**
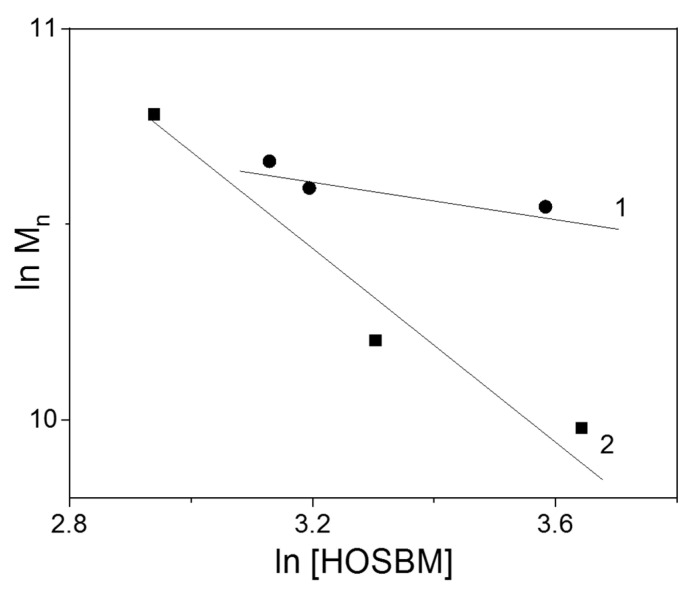
A log-log dependence of number average molecular weight (Mn) and HOSBM-BA-St (1)/HOSBM-BA-MMA (2) weight concentration.

In this work, obtained latexes were characterized by gel permeation chromatography (GPC) to evaluate their molecular weight and dispersity. Figure 4 shows the logarithmic dependence of number average molecular weight on the concentration of HOSBM (ln [HOSBM]) in monomer feed.

As expected, the molecular weight of the final latex polymers decreased as the fraction of POBMs in the initial monomer mixture (degree of unsaturation) increased. This is explained by the degradative chain transfer effect on HOSBM (allylic termination) provided by allylic hydrogen atoms in the HOSBM molecules, which was reported in our previous studies [19,20]. This observation was in agreement with the previously described more extensive chain transfer effect, caused by a higher unsaturation degree of the monomer feed. Interestingly, bimodal molecular weight distribution has been observed for latexes synthesized in the copolymerization of HOSBM and BA with MMA, while GPC analysis of material synthesized in copolymerization with St yields a single peak, although with broader dispersity (Figure S3). We explain this difference by various termination modes for macroradicals during chain copolymerization. The pathways of termination of MMA and St polymerizations are the most extensively studied [27]. The disproportionation (yielding two shorter macromolecules)/coupling ratio in MMA polymerization was 85/15, while for St polymerization the contribution of the coupling mode could be varied from nearly 60% up to 100%. According to the literature data [27], the GPC analysis of the MMA polymers revealed a bimodal trace which is divided into two components (low and high molecular weight) and confirms the mixed pathway of termination. For both compositions, though, the measurements clearly showed the presence of lower and higher molecular weight fractions, which is clearly beneficial for their adhesive performance.

**Figure 5:**
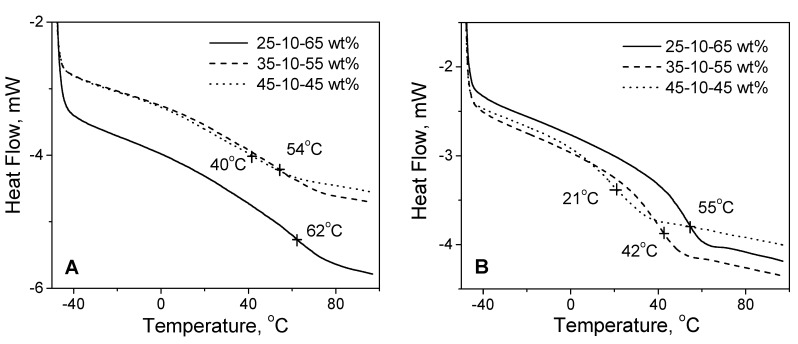
DSC diagrams for terpolymers HOSBM-BA-MMA (**A**) and HOSBM-BA-St (**B**) (heating rate: 10 °C min^−^^1^).

To evaluate the effect of HOSBM content on the thermal properties of plant oil-based latex adhesives, latex samples underwent differential scanning calorimetry (DSC) measurements. As illustrated in Figure 5, the DSC diagrams show a dependence of glass transition temperature (Tg) for synthesized latexes on different HOSBM content. The plasticizing effect of POBM fragments has been previously reported by our group [20,21]. The obtained results confirmed that Tg decreased when HOSBM content increased, making the resulting materials much softer if compared to polystyrene and polymethyl methacrylate (both having glass transition temperature in a range of 100–110 °C). A variation of oil-based monomer content changed the Tg (and, thus, the thermal properties) of the resulting latex polymers for all synthesized compositions. An increasing fraction of HOSBM in copolymers made the macromolecules more flexible, as indicated by decreasing Tg.

### 2.2. Correlation between Wettability and Adhesive Performance Plant Oil-Based Terpolymers

Good wettability of material surfaces is an important factor for enabling bonding formation between adhesive and substrate. It is critical that the applied adhesive formulation wets and spreads over the surface to achieve close contact between two materials to ensure physical interactions and the developing intermolecular forces.

The substrate surface properties, including surface energy and roughness, have an essential impact on the resulting bonding quality. Another surface characteristic that can be translated into adhesive performance is contact angle hysteresis (CAH). CAH is determined as a difference between advancing contact angle, *θ_A_* of the liquid that spreads over a surface, and receding contact angle, *θ_R_* of a liquid that retreats from a wet surface. Surface roughness, chemical heterogeneity, and surface reorganization upon close contact with a liquid during the measurement are considered to be the most influential factors for CAH values [28,29].

Hence, the next step in this study was to investigate how latex copolymer composition (including POBM content) impacts wettability (ability to maintain contact with substrate surface) of various material surfaces by latexes and if the latter correlates with the plant oil-based latexes adhesive performance. A range of substrates representing semicrystalline polyethylene terephthalate film (PET), polypropylene sheet (PP), bleached paperboard (uncoated), and a top coated with a clay mineral bleached paperboard were sourced for this purpose. Prior to studying the wettability of each material substrate by the synthesized latexes, surface energy for each substrate material was determined using the Zisman method [30,31]. According to this model, the surface free energy splits between dispersive and polar energy components, which can be calculated from the contact angles between the materials’ surface and probe liquids of different (known) interfacial properties (for example, water and diiodomethane).

As shown in Table 2, the surface energy of PP was below 35 mN/m, while the polar energy component was very low, which meant that PP was the most non-polar material in the range. Uncoated paperboard, with its polar functional groups of cellulose/hemicellulose, possesses slightly higher surface energy. Among chosen materials, the highest surface energy of PET can be attributed to the polar ester groups. However, the polar energy component of the PET surface was still lower than for the top coated with hydrophilic clay mineral paperboard substrate.

Table 3 presents experimentally determined surface free energy for each HOSBM-based latex synthesized in this work. The obtained values (ranging between 27 and 30 mN/m) do not depend significantly on copolymer composition (including biobased content), revealing that latexes possess rather low surface energy, which can be explained by the presence of remaining surfactant molecules at the liquid–air interface. Taking into account the experimental precision, no difference between latexes with various POBM content was observed.

To correlate surface properties and adhesive performance of HOSBM-based copolymer latexes and ensure latex spreading ability and adherence over the substrate materials differing by polarity, contact angle hysteresis for each latex-substrate pair was next determined using water as a contact liquid.

Table 3 and Table 4 collect contact angle hysteresis values for latexes with 25 and 45 wt. % of HOSBM on PP, PET, and coated/uncoated paperboard substrates. Even though the surface free energy of substrate materials differed in a range of 34–47 mN/m (with a polar energy component differing by one order of magnitude), no significant difference appeared for the experimentally determined values of hysteresis with increasing plant oil-based content in the copolymer. Nevertheless, essentially higher values for both advancing and receding contact angles were observed for coated/uncoated paperboard, compared to PP and PET substrates, which may indicate different adherence of latexes to both paperboard substrates and might be explained by more expressed chemical heterogeneity of the substrate surface [32].

Overall, wettability measurements indicated that the synthesized ternary copolymers latexes exhibited an ability to spread and adhere once applied to all chosen substrates, and, thus, could be considered further for adhesive performance evaluation. Therefore, the adhesive performance of latexes was studied next using the T-peel test.

Since PET, PP, and paperboard are widely used in food packaging, the textile industry in manufacturing carpets, woven materials, etc., as well as other consumer goods, T-peel strength testing can be applied for assessing the adhesive joints in laminated or packaging materials [33]. Having this in mind, the synthesized HOSBM-based copolymers were tested on PET to PP and coated to uncoated paperboard substrate pairs to demonstrate the latexes’ feasibility in multisubstrate bonding applications.

**Figure 6:**
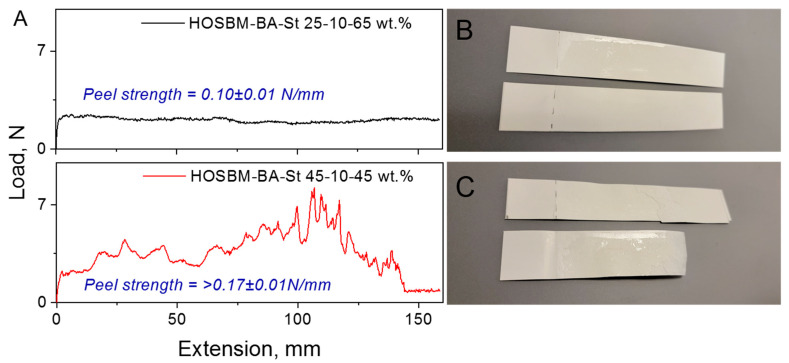
Peel strength of HOSBM-BA-St 25–10–65 wt. % and 45–10–45 wt. % latexes copolymers on paperboard (coated to uncoated) substrates (**A**) and substrate samples after peel strength testing ((**B**,**C**) [substrate failure]).

Typically, adhesion force is determined by wettability and the resulting physicochemical interactions between adhesive and substrate, including strong covalent bonding as well as weaker physical (van der Waals or hydrogen) interactions [24]. Considering the chemical structure of plant oil-based monomers, it can be assumed that the presence of oil-derived unsaturated fatty acid fragments in latexes may provide cross-linking sites, thus strengthening adhesion forces. Figure 6A shows two selected plots for HOSBM-BA-St latexes with different biobased content tested by T-peel test on paperboard (coated to uncoated) substrates. As seen qualitatively, cohesive failure was observed for latex with lower HOSBM content (Figure 6B), while the presence of 45 wt. % biobased content in the material resulting in higher peel strength and substrate failure during the testing (Figure 6C). In the case of cohesive failure, the peel plot stayed at a plateau during testing, and both substrates contained adhesive residues after the experiment ended. The peeling plot looked more complex for the latexes with higher biobased content. The load initially rose to a maximum point, then dropped to a lower value, corresponding to the noticeable delamination of the substrate (Figure 6C).

**Figure 7:**
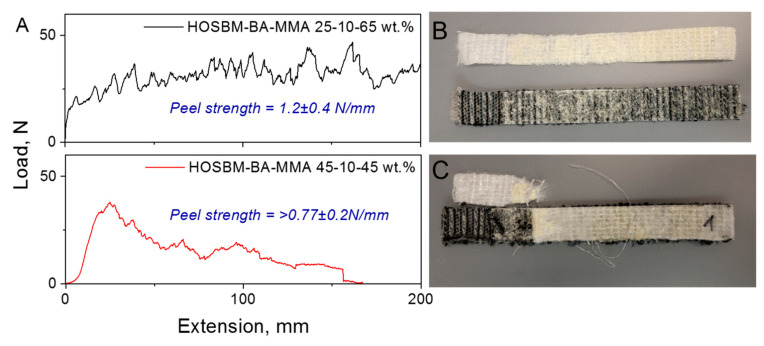
Peel strength of HOSBM-BA-MMA 25–10–65 wt. % and 45–10–45 wt. % latexes copolymers on PET-PP substrates (**A**) and substrates samples after T-peel strength testing ((**B**,**C**) [substrate failure]).

Peel strength of HOSBM-BA-MMA latexes with various biobased contents were tested on PET-PP substrate pairs (Figure 7). The obtained results showed that the presence of HOSBM fragments in the latex terpolymers (up to 45 wt. % in monomer feed) improved adhesion and increased the peel strength. Similar to the testing on paperboard, using latexes with lower biobased content resulted in cohesive failure, and substrate failure was observed once a higher concentration of biobased fragments was incorporated into latex copolymers. We attributed an increase in peel strength to the stronger chemical bonding formation at the terpolymer-substrate interface. As a result, substrate failure occurred due to the strong adhesion of the terpolymers with higher biobased content to the substrate, which was more substantial than the structural cohesion force of the latex (Figure S4). Therefore, it can be concluded that increasing the concentration of HOSBM fragments in the latex terpolymers led to differences in adhesive joint failure mode during peel strength testing (which was cohesive failure for a lower amount of HOSBM vs. substrate failure when the amount of HOSBM in the terpolymer went up).

The obtained results indicated that the adherence level of latexes overall correlated with wettability, thus contact angle hysteresis measurements reflected POBM-based ternary copolymers behavior on substrates with various chemical heterogeneities.

In summary, the performance of the synthesized latexes with up to 45 wt.% of high oleic soybean oil-based monomer showed their utility on multiple substrates, resulting in substrates’ failure in adhesive peel strength testing.

## 3. Materials and Methods

### 3.1. Materials

High oleic soybean oil (Perdue Agribusiness LLC, Salisbury, MD, US), sodium dodecyl sulfate (SDS, VWR; Solon, OH, US), and sodium chloride (VWR; Solon, OH, US) were used as received. Methyl methacrylate (MMA, Alfa-Aesar; Ward Hill, MA, USA), butyl acrylate (BA, TCI America, Portland, OR, USA), styrene (St, Sigma-Aldrich, St. Louis, MO, US), and acrylic acid (AA, Alfa-Aesar; Ward Hill, MA, USA) were distilled under vacuum to remove the inhibitor and stored in a refrigerator. Azobisisobutyronitrile (AIBN; Sigma-Aldrich, St. Louis, MO, USA) was purified with recrystallization from methanol. All other solvents used were reagent grade or better and used as received. Deionized water was used throughout the study (Millipore water, MilliQ, 18 MΩ).

### 3.2. Latex Preparation

The high oleic soybean oil-based latexes were synthesized in mini-emulsion copolymerization of respective acrylic monomers from plant oils with BA, St, and MMA. For this purpose, the oil phase (25.5 g) was prepared by mixing HOSBM (25–45 wt. %, 6.4–11.5 g), BA (10 wt. %, 2.55 g) with MMA or St at different ratios (40–55 wt. %, 11.45–16.55 g) in the presence of 1.5 wt. % (0.4 g) oil-soluble initiator per oil phase.

The aqueous phase was formed by dissolving the emulsifier (SDS, 5 wt. % per oil phase, 1.3 g) in Millipore water with added small amounts of NaCl (0.02 mol/L, 0.05 g). The oil phase was added dropwise to the aqueous phase and mixed for 45 min to form a pre-emulsion. The pre-emulsions were sonicated with three pulses for 60 sec each using Q-Sonica (500 W digital sonicator, ½ in. tip, 20 kHz, Newtown, CN, USA) and placed in an ice bath to maintain the temperature at 25 °C. The resulting miniemulsions were purged with nitrogen for 10 min and polymerized at 70 °C for 10 h. The latex solid content was kept at 30 wt. %.

### 3.3. Plant Oil-Based Latex Characterization

Total monomer conversion was determined by multiple precipitations of latex copolymers in methanol to remove residual unreacted monomers. The purified copolymers were dried in an oven until a constant weight was achieved.

The latex solid content was measured gravimetrically by drying the samples in an oven at an elevated temperature for 45 min.

The latex copolymers composition was analyzed using ^1^H NMR spectroscopy (JEOL ECA 400 MHz NMR Spectrometer, Akishima, Japan) using CDCl_3_ as a solvent.

The molecular weight of the latex copolymers was determined using gel permeation chromatography (GPC) (Waters Corporation Modular Chromatograph, which consists of a Waters’ 1515 HPLC pump, a Waters’ 2410 refractive index detector, and two 10 μm PL-gel mixed-B columns) at 40 °C using tetrahydrofuran as a carrier solvent.

Particle size distribution of the plant oil-based latex particles was measured using dynamic light scattering, Malvern Zetasizer Nano-ZS90 with a fixed scattering angle 90°, and a 633-nm wavelength laser. Samples were prepared by diluting one drop of latex in approximately 7 mL of water.

The glass transition temperature of POBM-based latex copolymers was determined by differential scanning calorimetry (DSC) (TA Instruments Q1000 calorimeter, New Castle, DE, USA) at heat/cool/heat mode (−50 °C/150 °C) with dry nitrogen purging through the sample at 50 mL/min flow rate. Latex sample’s heating/cooling rate was 10–20 °C/min.

### 3.4. Measurement of the Surface Free Energy, Surface Tension, Dynamic Contact Angles, and Contact Angle Hysteresis

The surface free energy of substrates, the surface tension of latexes, dynamic contact angles, and contact angle hysteresis of the latex coatings from plant oil-based adhesives were characterized using KRÜSS contact angle instruments DSA100 with an external tilting device PA3220.

Surface free energy, as well as the disperse and polar fractions of substrates, were determined according to the Zisman method. Water and diiodomethane were used as the standard test liquids. The surface free energy of substrates was measured at room temperature without pretreatment. For each sample, three to five contact angle measurements with water and diiodomethane were made at different locations.

The pendant drop method was used to determine the surface tension of synthesized latex terpolymers. In this method a latex drop of 4 μL volume was suspended from the syringe needle and allowed to stabilize for 5–10 min prior to surface tension measurement. Surface tension was calculated from the shadow image of a pendant drop using drop shape analysis. All measurements were conducted at room temperature.

Dynamic contact angles were measured as a drop moved across a tilted surface. Initially, the drop of the plant oil-based latex was deposited on a level substrate surface, then the table was slowly tilted and the inclination angle increased. For comparative measurements between different samples, the suitable measuring conditions (tilting speed = 60°/min, tilting position = 30°, and drop volume = 18–23 µL) were kept. Contact angle hysteresis was defined as the difference between the advancing (*θ_A_*) and receding (*θ_R_*) contact angles.

### 3.5. Adhesive Performance Testing

Peel strength of the POBM-based latexes was measured according to the ASTM D1876−08 method using MTS’s Insight Electromechanical 5 kN Extended Length Testing System with load cell 5 kN at a test speed of 304.8 mm/min.

#### 3.5.1. Peel Strength of PET-PP Substrates

The range of the synthesized latexes was applied on PET primary backing and polypropylene secondary backing. The test samples had a rectangular shape (length: 229 mm, width: 25 mm). The test panels were dried for 24 h at room temperature under a 4.5 kg weight press, then held at elevated temperature for 24 h, and cooled at standard conditions for 3 h.

The unbonded ends of the test specimen were clamped bent in the test grips of the tension testing machine. The load at a constant head speed of 304.8 mm/min was applied. The autographic recording of load versus distance peeled was made during the T-peel test. The peel resistance over at least a 127-mm length of the bond line after the initial peak was determined. The measurement of peel strength was repeated five times, and the average value was recorded.

#### 3.5.2. Peel Strength of Paperboard Substrates

A range of synthesized latexes was applied on paperboard substrate (bleached paperboard with a clay mineral coated top surface, 12 pt). The test samples had a rectangular shape (length: 152 mm, width: 25 mm). The tested panels were held at elevated temperature for 24 h and cooled at standard conditions for 3 h.

The unbonded ends of the test specimen were clamped bent in the test grips of the tension testing machine. The load at a constant head speed of 304.8 mm/min was applied. The peel resistance over at least a 100-mm length of the bond line after the initial peak was determined. The measurement of peel strength was repeated 5 times, and the average value was recorded.

## 4. Conclusions

Plant oil-based acrylic latex ternary copolymers with a range of performance capabilities and controlled Tg were developed and exhibited adhesive capabilities on various substrates. Adhesive performance of the latexes could be tailored by using different plant oil-based monomers in copolymerization, which might provide features required for specific applications. In this study, the best-performing latex adhesives containing up to 45 wt. % of high-oleic soybean oil-based monomer fragments demonstrated promising efficiency in the testing of PET to PP and coated to uncoated paperboard substrate pairs, resulting in substrate failure during the adhesive testing.

Since the incorporation of a higher amount of hydrophobic POBM in latex copolymers brings some limitations to the synthetic process, in the future, process parameters (solid content, surfactant amount, monomers feeding) need to be adjusted accordingly for different formulations.

## 5. Patents

Biobased Acrylic Monomers US 10,315,985 B2, 11 June 2019.

Biobased Acrylic Monomers and Polymers Thereof US 10,584,094 B2, 10 March 2020.

## Figures and Tables

**Table 1:** Solid content, conversion, and composition of terpolymer latexes.

Latex Formulations (wt. % in Monomer Feed)		Solid, %	Conv., %	Copolymers Composition, wt. %	
				Calculated	^1^H NMR
HOSBM-BA-MMA	25–10–65	30.3	94.2	0.18–0.05–0.77	0.19–0.06–0.75
	35–10–55	30.0	92.5	0.27–0.05–0.68	0.27–0.09–0.64
	45–10–45	30.4	90.5	0.36–0.05–0.59	0.39–0.08–0.53
HOSBM-BA-St	25–10–65	30.7	94.5	0.2–0.11–0.69	0.24–0.09–0.67
	35–10–55	30.9	93.0	0.28–0.11–0.61	0.26–0.11–0.63
	45–10–45	30.4	93.0	0.35–0.11–0.54	0.42–0.1–0.48

**Table 2:** Surface free energy parameters of the used materials.

Substrate	Water *θ*, °	CH_2_I_2_ *θ*, °	Surface Energy, mN/m		
			λ_S_^d^	λ_S_^p^	λ_S_
PET	73.4 ± 1.7	37.4 ± 1.3	40.9 ± 0.6	5.6 ± 0.7	46.5 ± 1.3
PP	93.1 ± 4.0	53.5 ± 2.9	32.3 ± 1.6	1.2 ± 0.8	33.5 ± 2.5
Paperboard coated	66 ± 3.4	58 ± 3.6	30 ± 2.1	12.8 ± 2.1	42.8 ± 4.2
Paperboard uncoated	83 ± 6.3	51 ± 2.6	33.9 ± 1.5	3.6 ± 2.1	37.5 ± 3.6

Water *θ*—water contact angle; CH_2_I_2_ *θ*—diiodomethane contact angle; λ_S_^d^—dispersive component of the surface energy; λ_S_^p^—polar component of the surface energy; λ_S_—surface free energy.

**Table 3:** Contact angle hysteresis of HOSBM-based latexes on PP and PET.

Sample (Monomer Feed)		Surface Tension, mN/m	PP			PET		
			*θ_A_,* Deg	*θ_R_,* Deg	*θ* *_A_–θ_R_*	*θ_A_,* Deg	*θ_R_,* Deg	*θ* *_A_–θ_R_*
HOSBM-BA-MMA	25–10–65	27.4	56 ± 1.4	25 ± 2.0	31	60 ± 1.2	29 ± 1.2	31
	45–10–45	27.7	61 ± 1.0	32 ± 1.8	29	60 ± 1.9	25 ± 1.7	35
HOSBM-BA-ST	25–10–65	28.9	65 ± 3.0	43 ± 3.2	22	62 ± 0.7	34 ± 0.6	28
	45–10–45	30.0	60 ± 2.5	34 ± 1.4	26	59 ± 1.0	31 ± 0.9	28

*θ_A_*—advancing contact angle; *θ_R_*—receding contact angle; *θ_A_*–*θ_R_*—contact angle hysteresis.

**Table 4:** Contact angle hysteresis of HOSBM-based latexes on coated/uncoated paperboard.

Sample (Monomer Feed)		Coated			Uncoated		
		*θ_A_,* Deg	*θ_R_,* Deg	*θ_A_*–*θ_R_*	*θ_A_,* Deg	*θ_R_,* Deg	*θ_A_*–*θ_R_*
HOSBM-BA-MMA	25–10–65	81 ± 1.9	49 ± 0.9	32	79 ± 1.3	49 ± 0.5	30
	45–10–45	80 ± 1.5	47 ± 1.0	33	82 ± 2.5	47 ± 0.5	35
HOSBM-BA-ST	25–10–65	80 ± 1.1	55 ± 1.5	25	83 ± 4.6	55 ± 3.1	28
	45–10–45	80 ± 0.8	49 ± 2.0	31	89 ± 1.6	59 ± 1.7	30

*θ_A_*—advancing contact angle; *θ_R_*—receding contact angle; *θ_A_*–*θ_R_*—contact angle hysteresis.

## Data Availability

Data is contained within the article.

## Supplementary Materials

The following supporting information can be downloaded at: https://www.mdpi.com/article/10.3390/molecules27165170/s1, Figure S1: Chemical structure of plant oil-based terpolymer HOSBM-BA-MMA with indicated integral proton shifts, Figure S2: ^1^H NMR spectrum of poly(HOSBM), Figure S3: Gel permeation chromatography (GPC) analysis diagram of terpolymers HOSBM-BA-MMA (A) and HOSBM-BA-St (B), Figure S4: T-peel adhesion strength testing on paperboard (A) and PP-PET (B) substrates after applying plant oil-based terpolymers.
