# Supplementary material for: Plant Oil-Based Acrylic Latexes towards Multisubstrate Bonding Adhesives Applications
Source: Molecules. 2022 Aug 13;27(16):5170. doi: 10.3390/molecules27165170 (PMC9416654; doi:10.3390/molecules27165170)
Supplement: Supplementary file 1 [file molecules-27-05170-s001.zip › molecules-1822375-supplementary.pdf]

Supporting Information

Plant Oil-based Acrylic Latexes Suitable for Multisubstrate Bonding Adhesives Applications

Vasylyna Kirianchuk,[1] Bohdan Domnich,[2] Zoriana Demchuk,[3] Iryna Bon,[2] Svitlana Trotsenko,[2] Oleh Shevchuk,[1] Ghasideh Pourhashem,[2] Andriy Voronov[2]*

[1] *Department of Organic Chemistry, Institute of Chemistry and Chemical Technologies, Lviv Polytechnic National University, Lviv, Ukraine, 79013*

[2] *Department of Coatings and Polymeric Materials, North Dakota State University, Fargo, ND, United States, 58102*

[3] *Chemical Sciences Division, Oak Ridge National Laboratory, Oak Ridge, TN, United States, 37830*

*Corresponding author. E-mail address: andriy.voronov@ndsu.edu

3 pages, 4 Figures

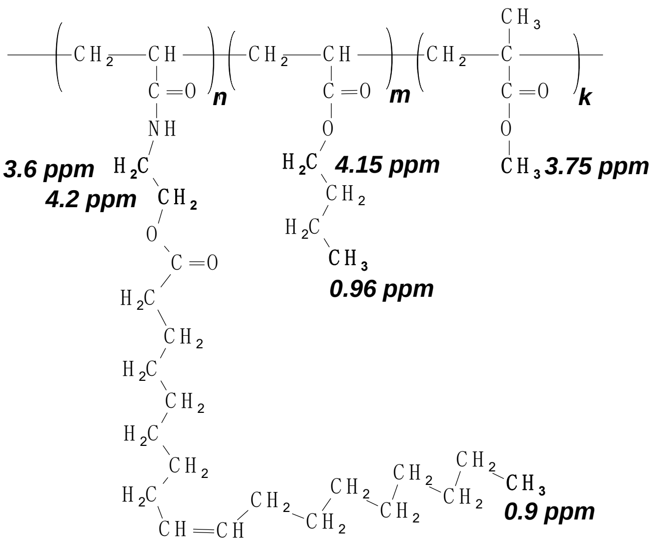

Figure S1. Chemical structure of plant oil-based terpolymer HOSBM-BA-MMA with indicated integral proton shifts

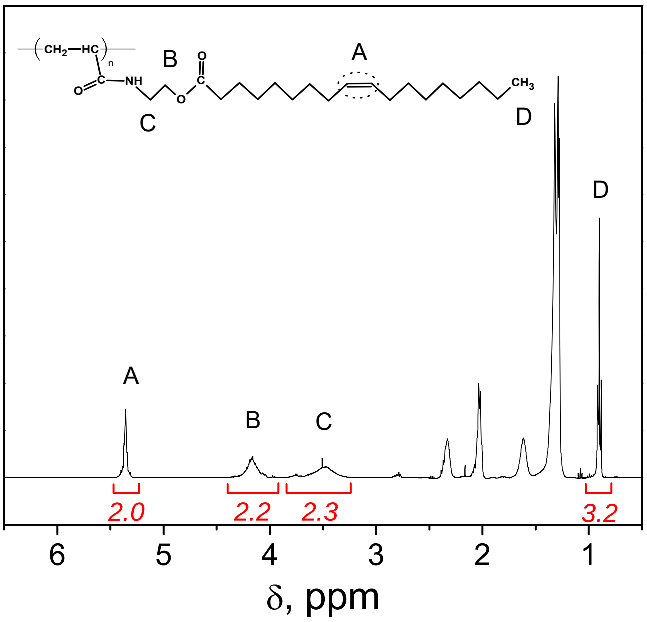

Figure S2. ^{1}H NMR spectrum of poly(HOSBM)

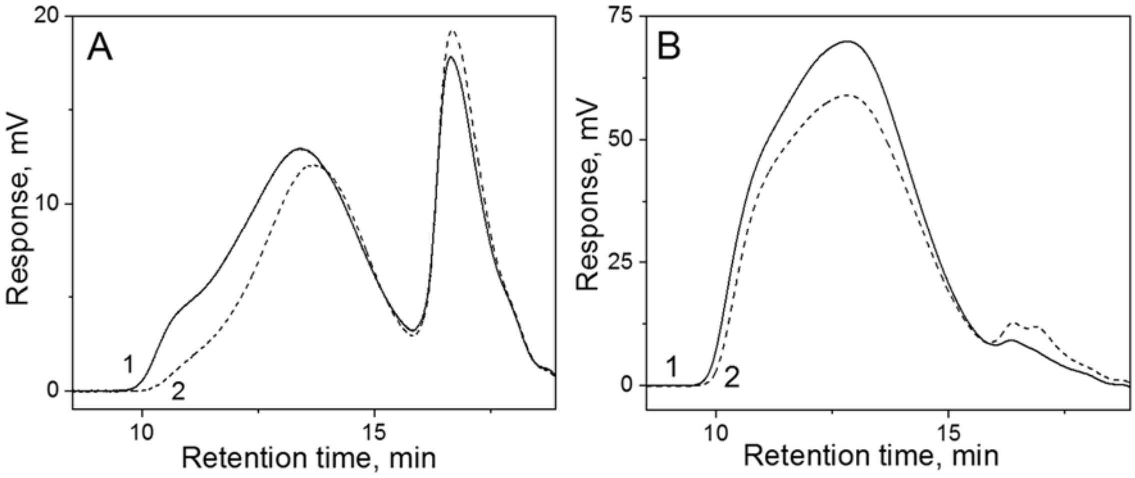

Figure S3. Gel permeation chromatography (GPC) analysis diagram of terpolymers HOSBM-BA-MMA (A) and HOSBM-BA-St (B)

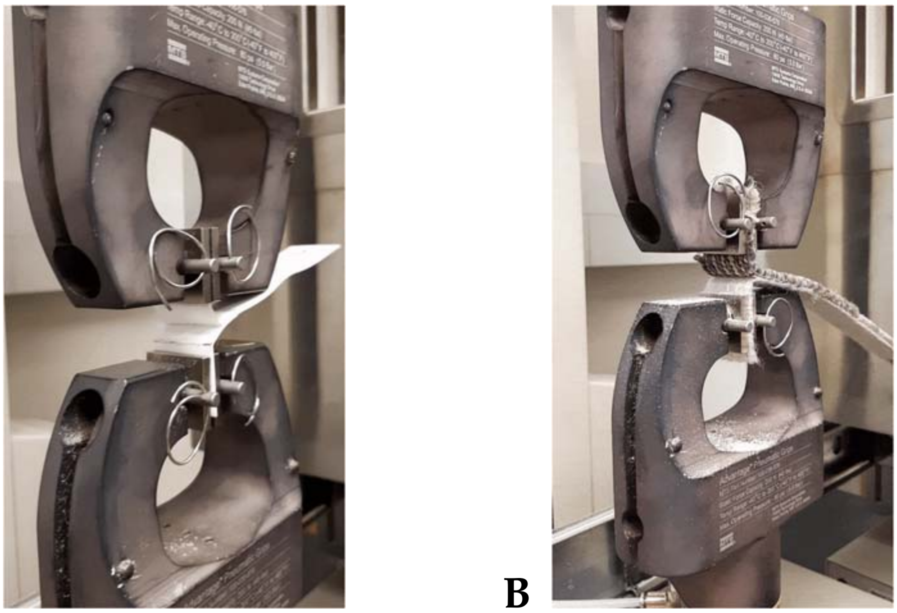

A B

Figure S4. T-peel adhesion strength testing on paperboard (A) and PP-PET (B) substrates after applying plant oil-based terpolymers
